# Interleukin-17A Orchestrates Lung Injury and Remodeling Through p53 and uPA System Crosstalk

**DOI:** 10.3390/ijms27041841

**Published:** 2026-02-14

**Authors:** Durgesh Nandini Das, Akarsha Balnadupete, Rashmi Shetty, Venkadesa Perumal Gopu, Rushil Sajjan, Yashodhar P. Bhandary, Amarnath S. Marudamuthu, Christian Oliver, Aarav Patel, Aryan Patel, Buka Samten, Yoichiro Iwakura, Hua Tang, Deborah E. Citrin, Jay Peters, Sreerama Shetty

**Affiliations:** 1 Texas Lung Injury Institute, Department of Cellular and Molecular Biology, The University of Texas Health Science Center at Tyler, 11937 US Highway 271, Tyler, TX 75708, USA; nandininitrkl@gmail.com (D.N.D.); akarsha.balnadupete@uttyler.edu (A.B.);; 2 Research Institute for Biomedical Sciences, Tokyo University of Science, Chiba 278-0022, Japan; 3 Radiation Oncology Branch, National Cancer Institute, National Institute of Health, Bethesda, MD 20892, USA; 4 Department of Medicine, Division of Pulmonary Diseases & Critical Care Medicine, The University of Texas Health Science Center at San Antonio, 4502 Medical Dr., San Antonio, TX 78229, USA

**Keywords:** IL-17A, anti-IL-17A/IL-17RA, CSP7, lung fibrosis, alveolar epithelial cells

## Abstract

Alveolar inflammation, elevated interleukin-17A (IL-17A), and fibrin deposition are common features in all forms of lung injury followed by fibrotic repair. Type II alveolar epithelial cell (AEC) viability, regulated by tumor suppressor protein p53 and changes in uPA-mediated fibrinolysis, has been linked to lung injury and pulmonary fibrosis (PF). Nevertheless, mechanistic details linking increased IL-17A with p53 and PAI-1 to lung injury and remodeling remain unclear. We found that IL-17A and its receptor (IL-17RA) are induced during various lung injuries. IL-17A augments IL-17RA, p53 and downstream PAI-1 with a concurrent decrease in uPA and its receptor (uPAR) in AECs. These changes promote AEC apoptosis, alveolar injury and PF. In addition, IL-17A causes a dose-dependent increase in IL-17RA and profibrogenic markers in lung fibroblasts (LFs), suggesting myofibroblast differentiation. We further found that inhibition of IL-17A by caveolin-1 scaffolding domain peptide (CSP) or its 7-mer deletion fragment (CSP7) inhibits AEC apoptosis, lung inflammation, and profibrogenic markers in LFs and PF. Further, treatment of mice with bleomycin-induced lung injury using CSP7, an anti-IL-17A antibody, or an IL-17RA blocking antibody attenuates total lung hydroxyproline and soluble collagen content, as well as levels of profibrogenic markers. These observations support the role of IL-17A/IL-17RA signaling in lung injury and post-injury remodeling.

## 1. Introduction

Acute and chronic lung injuries result in alveolar epithelial cell (AEC) death, abnormal fibrin turnover and persistent inflammation. The differentiation of type I AECs from facultative progenitor type II AECs plays a crucial role in normal post-injury alveolar epithelial repair. The lack of proper alveolar re-epithelialization after acute and chronic lung injuries due to the loss of AEC renewal resulting from excessive apoptosis and senescence promotes profibrotic repair. Multiple publications from our laboratory [1,2,3,4,5,6,7] and others [8,9] show that increased expression of the tumor suppressor protein and plasminogen activator inhibitor-1 (PAI-1) and the suppression of urokinase-type plasminogen activator (uPA) and uPA receptor (uPAR) expression promote pathways that lead to AEC senescence and apoptosis. Thus, reduced AEC viability and loss of renewal capacity potentiate fibrogenesis and progressive pulmonary fibrosis (PF) through the activation and differentiation of lung fibroblasts (LFs), as well as the expansion of activated myofibroblasts. Published reports from our group and others reveal that increased expression of p53 and its downstream target (PAI-1) results in senescence and apoptosis in the AECs of patients with chronic lung diseases, such as idiopathic PF (IPF) and chronic obstructive pulmonary disease (COPD), as well as in mice exposed to tobacco smoke (TS), bleomycin (BLM), silica, and thoracic radiation (TR)-induced early lung injuries or late PF [1,2,3,6,10,11,12,13,14,15,16,17,18,19].

A key contributor to these processes is interleukin-17 (IL-17), a cytokine mainly produced by T helper 17 (Th17) cells, a subset of CD4^+^ T cells that produce IL-17 family cytokines. IL-17 plays a dual role; it is an important driver of adaptive immunity against extracellular bacterial and fungal infections and modulates innate immune responses by influencing macrophage, neutrophil, and epithelial cell responses. Thus, IL-17 is implicated in various inflammatory disease processes, including autoimmune diseases [20,21]. The IL-17 cytokine family consists of IL-17A, IL-17B, IL-17C, IL-17D, IL-17E and IL-17F, and their immune responses are initiated by binding to their heterodimeric receptors IL-17RA, IL-17RB, IL-17RC, IL-17RD and IL-17RE [21,22]. Among the IL-17 family of cytokines, IL-17A and IL-17F share the highest (~50%) sequence homology [23,24,25,26,27]. However, these cytokines function differently due to differences in cellular sources, induction of pro-inflammatory mediators, and the distribution of their receptors [25,26]. IL-17F mainly contributes to the regulation of mucosal immunity [28,29] while IL-17B, IL-17C and IL-17D are involved in the induction of pro-inflammatory cytokines [27,30,31,32] and neutrophil recruitment but not epithelial effects [33,34]. IL-17E (aka IL-25) is involved in promoting Th2 immune responses [35,36]. IL-17A is involved in pro-inflammatory responses and plays a crucial role in defense against bacterial and fungal infections [37,38]. Cytokines such as IL-17A can directly target epithelial cells, including AECs and others, to produce antimicrobial responses and promote tissue remodeling [38]. Although IL-17A and IL-17F are vital in host defense, uncontrolled IL-17 responses aggravate inflammation and tissue damage [39]. Among the six members of the IL-17 cytokine family, IL-17A is closely associated with chronic lung diseases, such as IPF and COPD. IL-17A is produced by T-lymphocytes, macrophages and mast cells. IL-17A levels are markedly elevated in BAL and lung tissues of patients with IPF and COPD [40,41,42]. The pulmonary IL-17A level is also significantly elevated in various mouse models of experimentally induced lung injuries and PF, and IL-17A alone augments lung injury. AECs and LFs are targets of IL-17A, as they express its receptor, IL-17RA. Multiple studies have proven the critical role of IL-17A, particularly the IL-17A/IL-17RA signaling axis, in lung injury and lung remodeling [43,44,45]. Further, neutralization of IL-17A was effective in reducing lung injury and fibrosis in animal models [46,47]. However, the mechanisms by which IL-17A induces lung injury and remodeling are not well defined.

Mechanistically, IL-17A induces p53 in alveolar epithelial cells via IL-17RA-dependent signaling, leading to DNA damage responses and stabilization of p53. Activated p53 upregulates plasminogen activator inhibitor-1 (PAI-1) while suppressing urokinase-type plasminogen activator (uPA) and its receptor (uPAR), disrupting the uPA/PAI-1 balance. Elevated PAI-1 promotes epithelial senescence and apoptosis, limits fibrin turnover, and stabilizes the extracellular matrix, while reduced uPA and uPAR impair AEC proliferation. Together, these changes compromise AEC renewal, creating a profibrotic microenvironment that activates lung fibroblasts, drives fibrotic lung (myo)fibroblast (fLf) differentiation, and promotes excessive extracellular matrix (ECM) deposition, culminating in alveolar injury and progressive pulmonary fibrosis.

The hallmarks of chronic lung diseases, such as emphysema and PF, are alveolar injury and a lack of epithelial regeneration due to the loss of AEC renewal capacity, leading to the consequent activation, proliferation, and expansion of f LFs and the excessive production and deposition of collagen and other ECM proteins. These events culminate in the progressive obliteration of alveolar units by scar tissue and loss of lung function. Although evidence of the possible involvement of IL-17A in chronic lung diseases, including fibrotic lung diseases, is beginning to accumulate, there is limited knowledge regarding the role of the IL-17A/IL-17R axis in p53-mediated induction of AEC injury and progression of PF. In addition, it is unclear whether pleiotropic antifibrotic agents that attenuate lung injury and PF caused by particulates such as TS, silica, or BLM can be effective against IL-17A-induced lung injury. Therefore, to elucidate the molecular mechanisms by which IL-17A promotes AEC injury and PF, as well as to evaluate the potential therapeutic benefits of novel interventional approaches, we conducted studies using primary AECs, LFs, f LFs and lung homogenates. Additionally, we used tissues from normal individuals, COPD and IPF patients, as well as from naïve mice and mouse models of TSE-, BLM-, silica-, and TR-induced lung injury and PF, to better understand the molecular mechanism of IL-17A. To compare the responsiveness of AECs, LFs and f LFs to recombinant IL-17A and caveolin-1 (Cav1) scaffolding domain peptide (CSP) or CSP7 treatment, we used in vitro and in vivo approaches, including transgenic mice and archived human lung tissues.

## 2. Results

### 2.1. Increased IL-17A Levels in Lung Tissues of Patients with IPF and COPD and Mice with BLM-, TSE-, Silica- and TR-Induced Lung Injuries

**Figure 1:**
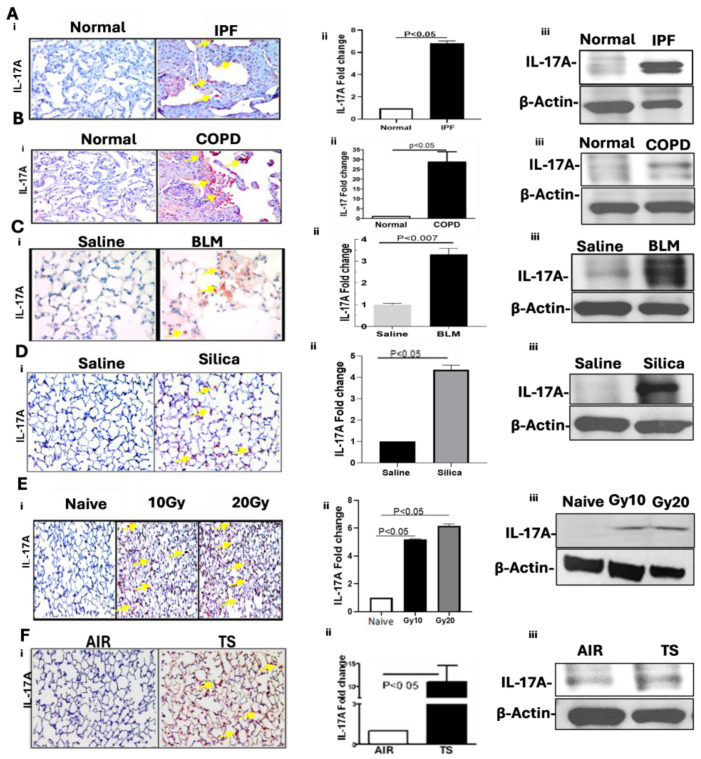
Increased IL-17A in lung diseases. (**A**) Human non-IPF (“normal”) lung (nL) tissue sections from control (non-IPF) donors and fibrotic lung (fL) tissues from patients with IPF were subjected to (**i**) IHC analyses for IL-17A antigen in situ. (**ii**) Total RNA from nL and fL (IPF) lung tissues was analyzed for IL-17A mRNA by qPCR. (**iii**) Homogenates of nL and fL (IPF) tissues were analyzed for IL-17A protein by Western blotting (WB). (**B**) Human nL (non-COPD) and COPD lung tissues were subjected to (**i**) IHC, (**ii**) qPCR and (**iii**) WB as above for IL-17A. (**C**) Lung sections (**i**) and total RNA (**ii**) or total protein (**iii**) from whole lung homogenates of control mice exposed to saline or mice with 1X-BLM-LI were analyzed for IL-17A, as described in (**A**). (**D**) Lung sections (**i**), total RNA (**ii**) and total protein (**iii**) from control (saline) mice or mice with silica-induced lung injury were evaluated for IL-17A. (**E**) Lung sections (**i**), total RNA (**ii**) and total protein (**iii**) from naïve control mice or mice exposed to TR (10 Gy or 20 Gy) were analyzed for IL-17A. (**F**) Lung sections (**i**), total RNA (**ii**) and total protein (**iii**) from control mice kept in ambient air or from mice with TSE-LI were evaluated for IL-17A. IL-17A mRNA levels were normalized with the corresponding level of GAPDH mRNA in all the qRT-PCR analyses. β-actin level was analyzed to assess loading equality in all WB.

IL-17A and its receptor IL-17RA are known to orchestrate crosstalk between the adaptive and innate immune systems during lung injuries. We therefore subjected lung sections from patients with IPF to IHC analyses for IL-17A expression. Consistent with the literature, IHC analyses revealed increased staining for IL-17A antigen in IPF lung tissues compared to similarly stained non-IPF control donor lung sections (Figure 1(Ai)). Quantitative real-time PCR (qRT-PCR) analyses of total RNA extracted from the lung homogenates confirmed significant elevation of IL-17A transcript levels in IPF lung tissues (Figure 1A(ii)). In agreement with the IHC and qPCR data, immunoblotting of lung homogenates from IPF tissues demonstrated a marked increase in IL-17A protein, further corroborating the upregulation of IL-17A in diseased lungs (Figure 1(Aiii)). We next analyzed COPD and non-COPD control donor lung sections for changes in IL-17A expression by IHC and found intense staining for IL-17A antigen in COPD lung sections compared to that in the control donor lung section (Figure 1(Bi)). Consistent with intense staining of IL-17A antigen in COPD tissues, qPCR analysis of total RNA (Figure 1(Bii)) verified a significant increase in IL-17A mRNA levels in COPD lung tissues versus donor (non-COPD) controls. Similarly, Western blotting of lung homogenates (Figure 1(Biii)) confirmed elevated IL-17A protein expression in COPD lungs. These findings validate the marked elevation of IL-17A in lung tissues during chronic lung diseases such as IPF and COPD.

Because the IL-17A level is markedly increased in IPF and COPD lung tissues, we next investigated whether IL-17A is similarly upregulated in four different preclinical mouse models of lung injury induced by BLM, TSE, silica and TR. As shown in Figure 1(Ci), lung sections from mice with BLM-LI exhibited intense IL-17A staining. These observations were corroborated by qPCR analysis of total RNA for IL-17A transcripts (Figure 1(Cii)) and immunoblotting of protein extract for IL-17A protein (Figure 1(Ciii)) from the whole lung homogenates of mice with 1X-BLM lung injury. We and others have previously reported that inhalation of silica causes acute lung injury and subsequent PF through elaboration of p53 crosstalk with major components of the uPA fibrinolytic system [10,16]. Therefore, we analyzed lung sections of mice exposed to silica and detected increased staining of IL-17A antigen in mice with silica-induced lung injury versus naïve control mice (Figure 1(Di)). This was further supported by a significant increase in IL-17A mRNA (Figure 1(Dii)) and IL-17A protein levels (Figure 1(Diii)). In addition, lung injury due to TR caused a dose-dependent increase in IL-17A antigen levels (Figure 1(Ei)), IL-17A mRNA (Figure 1(Eii)) and IL-17A protein (Figure 1(Eiii)), as assessed by IHC analysis of lung sections and qPCR and Western blot of whole lung homogenates, respectively. As IL-17A protein and IL-17A mRNA are markedly increased in COPD lungs versus control (non-COPD) lung tissues, and TSE induces AEC apoptosis and distal lung damage [7], we sought to determine whether IL-17A level is similarly increased in mice with TSE-LI. IHC analysis of lung sections of mice with TSE-LI showed a marked increase in IL-17A antigen staining (Figure 1(Fi)). This was confirmed by a significant increase in IL-17A mRNA (Figure 1(Fii)) and IL-17A protein (Figure 1(Fiii)) compared to the baseline level of IL-17A detected in control mice kept in ambient air.

### 2.2. Role of IL-17A in BLM-LI and TSE-LI

**Figure 2:**
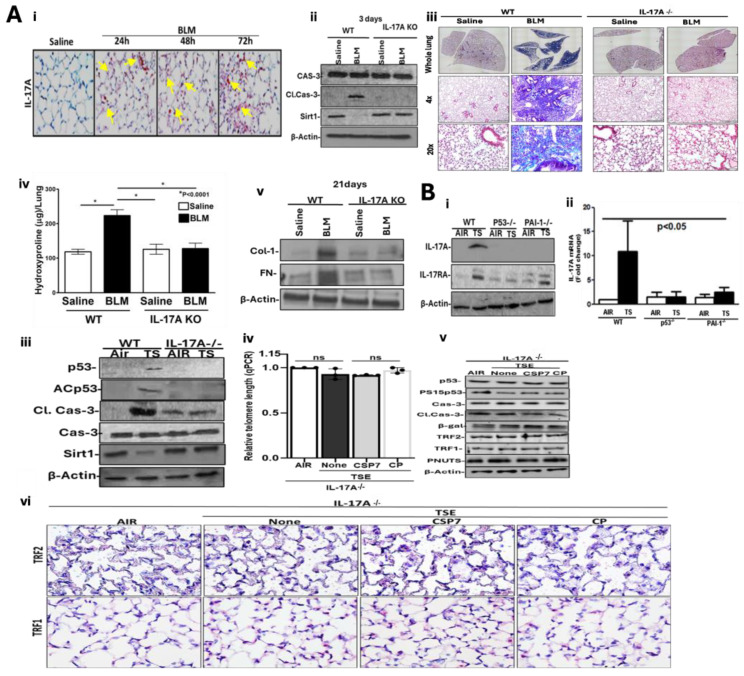
Regulation of BLM-LI and TSE-LI by IL-17A. (**A**) Lung sections of WT mice exposed to saline or BLM for 24–72 h were subjected to IHC staining for IL-17A antigen (**i**). AECs isolated from WT and IL-17A-deficient mice exposed to saline or BLM were analyzed for apoptosis and Sirt1 expression by Western blotting (WB) three days later (**ii**). Lung sections of WT and IL-17A^−/−^ mice exposed to saline or BLM were subjected to Masson’s trichrome staining 21 days later (**iii**). Whole lung homogenates of WT and IL-17A^−/−^ mice exposed to saline or BLM as in were analyzed for total HYP content (**iv**) or for Col-1 and FN by WB 21 days after BLM (**v**). (**B**) WT, p53^−/−^ and PAI-1^−/−^ mice were kept in ambient air or exposed to passive TS for 20 weeks. Total lung homogenates of control mice kept in ambient air, TSE WT mice, and p53^−/−^ or PAI-1^−/−^ mice were analyzed for IL-17A and IL-17RA protein by WB (**i**). Total RNA from mice kept in ambient air, TSE WT mice, and p53^−/−^ or PAI-1^−/−^ mice was analyzed for IL-17A mRNA (**ii**). AECs from TSE WT and IL-17A^−/−^ mice were analyzed for p53, ACp53, cleaved (Cl.) caspase-3/caspase-3 and Sirt1 by WB (**iii**). AECs from lungs of IL-17A^−/−^ mice kept in ambient air or TSE, as well as mice treated with or without CSP7 or CP, were analyzed for telomere length by qPCR (**iv**). AECs from lungs of IL-17A^−/−^ mice treated as in (**B(iv**)) were immunoblotted for the listed proteins (**v**). Lung sections of IL-17A^−/−^ mice treated as in (**B**(**iv**)) were subjected to IHC for telomere binding shelterin complex (TRF1 and TRF2) proteins (**vi**).

We and others have previously reported significant lung inflammation due to pulmonary influx of inflammatory cells during BLM-LI, TSE-LI and other lung injuries in mice [3,4,5,6,10,11,15]. IL-17A is a major cytokine that promotes neutrophil influx and activation through induction of chemokines such as CXCL1/GRO-α, IL-8/CXCL8 and MIP-1b/CCL4 [48,49]. Therefore, we stained lung sections of mice with BLM-LI for IL-17A antigen. As shown in Figure 2(Ai), pulmonary IL-17A staining increased from the minimal baseline level within 24 h after BLM exposure and peaked through 72 h, coinciding with maximum induction of p53 and apoptosis in AECs [6].

Because p53-mediated induction of PAI-1 contributes to AEC injury and mice deficient in p53 or PAI-1 resist early lung injury [3,4], we isolated AECs from WT and IL-17A-deficient mice 3 days after exposure to saline or BLM and tested for differences in apoptosis. Consistent with our earlier reports [6] and peak induction of IL-17A in lung tissues of WT mice (Figure 2(Ai)), AECs of WT mice analyzed 3 days post-BLM exhibited an increase in activation of caspase-3 (cleaved caspase-3) with suppression of histone deacetylase, Sirt1, compared to saline-treated control mice (Figure 2(Aii)), suggesting that increased p53-miR-34a feedback contributes to AEC apoptosis. However, BLM exposure failed to induce cleaved caspase-3 or inhibit Sirt1 from the basal level in AECs isolated from mice lacking IL-17A. These findings suggest that IL-17A promotes AEC apoptosis through p53-miR-34a-mediated inhibition of Sirt1. Since BLM-LI promotes subsequent remodeling, we analyzed whole lung homogenates of WT and IL-17A-deficient mice exposed to saline or BLM 21 days after inception of BLM-LI in WT mice for differences in progressive PF. As shown in Figure 2(Aiii), trichrome staining demonstrated a marked increase in collagen and other ECM protein deposition in the lungs of WT mice with BLM-LI in situ. However, trichrome-stained lung sections of IL-17A-deficient mice showed minimal ECM protein accumulation. This was consistent with a significant increase in total hydroxyproline (HYP) content in the whole lung homogenates of WT mice exposed to BLM compared to the total lung HYP content found in saline-treated control mice (Figure 2(Aiv)). However, consistent with the resistance of IL-17A-deficient mice to early lung injury, BLM failed to augment total lung HYP content in these mice, suggesting that IL-17A induced during BLM-LI contributes to the subsequent development of PF. This was further confirmed by increased levels of matrix proteins such as collagen-1α1 (Col-1) and fibronectin (FN) content in the whole lung homogenates of BLM-treated WT mice compared to the corresponding levels in IL-17A-deficient mice exposed to BLM, saline-treated WT or IL-17A-deficient control mice (Figure 2(Av)) 21 days after exposure.

Since IL-17A increased in the lungs of WT TSE-LI mice while p53- and PAI-1-deficient mice resisted TSE-LI, we analyzed the lung tissues of TSE WT, p53^−/−^ and PAI-1-/- mice for induction of IL-17A and its receptor, IL-17RA. As shown in Figure 2(Bi), Western blot analysis of lung homogenates from WT mice with TSE-LI revealed elevated IL-17A and IL-17RA protein levels. qPCR analysis of total lung RNA further depicted elevated IL-17A mRNA in WT TSE-LI mice (Figure 2(Bii)) compared to control mice maintained in ambient air. In contrast, TSE failed to induce IL-17A or IL-17RA protein or IL-17A mRNA in p53- or PAI-1-deficient mice. In addition, a concurrent increase in IL-17A and IL-17RA in lung tissues of TSE-LI WT mice suggests that IL-17A induces its cognate receptor expression. TSE augments IL-17A and IL-17R in lung tissues in WT mice, which is resisted by p53- and PAI-1-deficient mice. Therefore, we exposed WT and IL-17A-deficient mice to 20 weeks of TS, and AECs isolated from these mice were tested for p53, ACp53, cleaved caspase-3 and Sirt1 expression. In agreement with BLM-LI, TSE-LI caused a marked increase in activated (cleaved) caspase-3 with parallel induction of p53 and ACp53 and inhibition of baseline Sirt1 expression compared to control mice kept in ambient air, suggesting that p53-miR-34a-mediated inhibition of Sirt1 contributes to AEC injury. However, TSE failed to induce p53 or ACp53 or apoptosis or inhibit the expression of Sirt1 from the baseline level otherwise observed in naive WT kept in ambient air or IL-17A-deficient mice (Figure 2(Biii)). The pathogenesis of COPD and IPF is often associated with telomere dysfunction and premature cellular aging. Our recent findings demonstrate significant telomere shortening and changes in shelterin complex binding protein due to increased p53-uPA system crosstalk [15]. This was attenuated following treatment of TSE-LI mice with CSP7. Therefore, we isolated AECs from IL-17A-deficient mice exposed to 20 weeks of TS and analyzed them for telomere dysfunction. As shown in Figure 2(Biv), TSE failed to induce significant telomere shortening in AECs of mice deficient in IL-17A expression. Consistent with resistance to telomere dysfunction and TSE-LI, CSP7 or CP had minimal effect in these mice. This was further confirmed by minimal induction of p53 and P15Sp53, active caspase-3, or β-galactosidase (β-Gal) protein in AECs of TSE IL-17A-deficient mice versus those kept in ambient air. Further, TSE failed to affect the baseline expression of shelterin binding proteins such as TRF1, TRF2 and PNUTS in AECs of IL-17A-deficient mice (Figure 2(Bv)), suggesting that IL-17A deficiency and resistance to telomere shortening, apoptosis, and senescence in AECs and TSE-LI are intricately linked. IHC analysis of lung sections further revealed little change in TRF1 or TRF2 antigen staining in IL-17A-deficient mice subjected to TSE versus those kept in ambient air (Figure 2(Bvi)). Because these mice resist TSE-LI and telomere shortening, CSP7 treatment had minimal effects in these mice, unlike WT mice [15].

### 2.3. T Cell-Derived IL-17A: Roles of CD4^+^ and CD8^+^ Subsets

**Figure 3:**
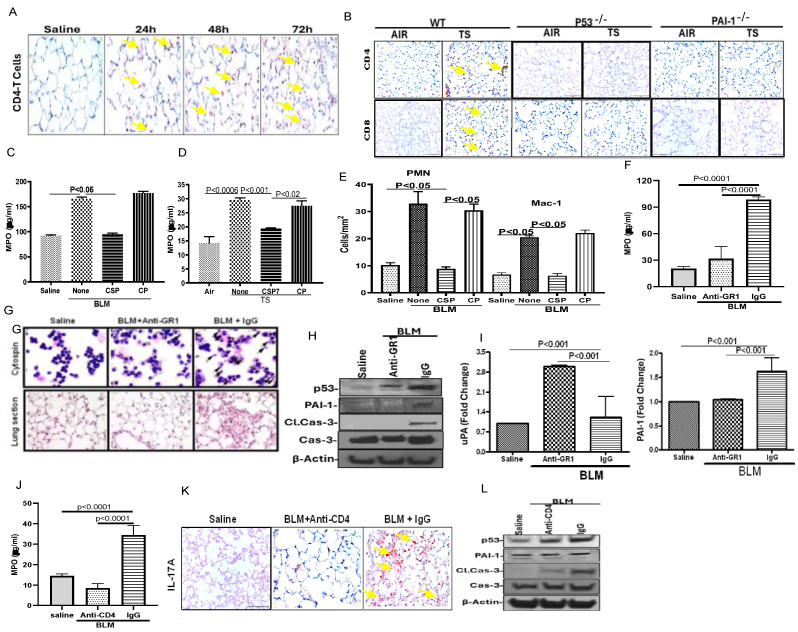
Accumulation of CD4^+^ and CD8^+^ T cells in lungs of mice during BLM-LI and TSE-LI. (**A**) Lung sections of WT mice exposed to saline or BLM (for 24–72 h) were subjected to IHC staining for CD4^+^ T cells. (**B**) Lung sections of WT or p53^−/−^ and PAI-1^−/−^ mice exposed to TS were subjected to IHC staining for CD4^+^ and CD8^+^ T cells. (**C**) CSP harboring the CSP7 sequence inhibits MPO activity in WT mice with BLM-LI. (**D**) CSP7 inhibits MPO activity in WT mice with TSE-LI. (**E**) CSP attenuates pulmonary influx of PMNs and macrophages in mice with BLM-LI. (**F**) Inhibition of MPO activity in BLM-LI mice pretreated with anti-GR1 antibody. (**G**) Staining of BAL cells and lung sections of WT BLM-LI mice pretreated with anti-GR1 antibody. (**H**) Whole lung homogenates of WT mice with BLM-LI pretreated with anti-GR1 antibody were analyzed for p53, PAI-1 and activated caspase-3 by Western blotting (WB). (**I**) qPCR analyses of total lung RNA from WT BLM-LI mice pretreated with anti-GR1 antibody for uPA and PAI-1 mRNA. (**J**) Inhibition of MPO activity in BLM-LI mice pretreated with anti-CD4 antibody. (**K**) IHC of lung sections of WT BLM-LI mice pretreated with anti-CD4 antibody for IL-17A. (**L**) Whole lung homogenates of WT BLM-LI mice pretreated with anti-CD4 antibody were analyzed for p53, PAI-1 and activated caspase-3 by WB.

The literature suggests that IL-17A is mainly produced by CD4^+^, CD8^+^ and γδ T cells, which are increased in the lungs of mice with BLM-LI and TSE-LI. We therefore subjected lung sections of mice with BLM-LI for IHC using anti-CD4 antibody (Figure 3A). Consistent with an increase in IL-17A levels, an increase in pulmonary influx of CD4^+^ staining was apparent in WT mice between 24 and 72 h after BLM exposure. Like BLM-LI, TSE-LI augments p53 and PAI-1, and mice deficient in p53 or PAI-1 resist BLM-LI and TSE-LI. We therefore subjected lung sections from WT, p53^−/−^ and PAI-1^−/−^ TSE mice to IHC using anti-CD4 and CD8 antibodies (Figure 3B). Consistent with increased IL-17A staining in TSE-LI in WT mice, lung sections from WT mice with TSE-LI also showed an increase in CD4^+^ and CD8^+^ staining, indicating the influx of T-lymphocytes. This was, however, resisted by p53^−/−^ and PAI-1^−/−^ mice, suggesting induction of p53 and downstream PAI-1 due to TSE contributing to pulmonary influx of CD4^+^ and CD8^+^ T cells as well as IL-17A and lung injury. Since the IL-17A elaborated during BLM-LI and TSE-LI can induce inflammation by attracting neutrophils and other inflammatory cells, we analyzed the lung tissues of mice with BLM-LI (Figure 3C) or TSE-LI (Figure 3D) for myeloperoxidase (MPO) activity and found that MPO activity was significantly increased in the lung tissues of BLM-LI and TSE-LI mice, suggesting ongoing lung inflammation. This was significantly reduced after treatment of BLM-LI or TSE-LI mice with CSP or CSP7, whereas control mice exposed to CP showed elevated MPO activity. Since PMNs and macrophages contribute to lung inflammation and injury, we subjected BAL cells to differential staining and found a significant increase in BAL PMN and macrophage counts in mice three days after BLM-LI (Figure 3E). However, treatment of WT mice with CSP 24 h after BLM significantly reduced both PMN and macrophage counts, unlike mice with BLM-LI exposed to CP. PMN level is significantly elevated in mice with BLM-LI, and inhibition of BLM-LI by CSP is also associated with significant suppression of pulmonary PMN levels. Therefore, we next sought to test whether the anti-GR1 antibody can prevent BLM-LI and inflammation in WT mice. We found that pretreatment of mice with anti-GR1 antibody, 12 h and 2 h before BLM, significantly reduced MPO activity (Figure 3F) and depleted accumulation of PMNs in the lungs (Figure 3G) compared to control mice exposed to control antibody (IgG) and BLM. Further analysis of AECs isolated from WT mice exposed to anti-GR1 antibody and BLM demonstrated marked inhibition of BLM-induced p53, PAI-1, and active caspase-3, which were otherwise elevated in AECs of WT mice exposed to control antibody and BLM (Figure 3H). As shown in Figure 3I, anti-GR1 pretreatment increased expression of uPA mRNA and reduced PAI-1 transcript otherwise altered due to BLM-LI. This suggests that increased influx of PMN contributes to AEC death and lung injury through induction of p53 and p53-mediated downstream changes in uPA and PAI-1 expression.

Further literature suggests that CD4^+^ T cells are the main source of IL-17A. We next depleted CD4^+^ T cells of mice using an anti-CD4 antibody and treated mice with BLM. Three days after BLM, lung sections of WT mice pretreated with three (−3, 0, and 3 day) doses of anti-CD4 antibody, as well as whole lung homogenates, were assayed for MPO activity. As shown in Figure 3J, pretreatment of WT mice with anti-CD4 antibody caused a significant reduction in MPO activity compared to the levels in the lungs of control antibody and BLM-treated mice. IHC analysis of IL-17A expression in lung sections of WT mice pretreated with anti-CD4 antibody indicated a marked reduction in BLM-induced IL-17A compared to control mice exposed to control IgG and treated with BLM (Figure 3K). Immunoblotting of isolated AECs for p53, PAI-1 and cleaved caspase-3 indicated marked suppression compared to control IgG-pretreated mice exposed to BLM, suggesting that increased IL-17A produced by pulmonary influx of CD4^+^ cells at least partly contributes to lung injury and AEC apoptosis through induction of p53 and PAI-1 (Figure 3L).

### 2.4. CSP Attenuates IL-17A Levels in Lungs of WT Mice with TSE-LI and BLM-LI

**Figure 4:**
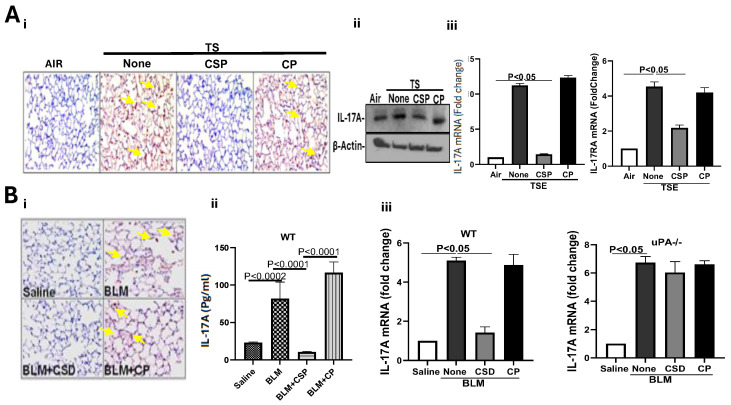
CSP inhibits IL-17A in the lungs of WT mice with TSE-LI and BLM-LI. (**A**) CSP inhibits IL-17A in the lungs of WT mice with TSE-LI. Sixteen weeks after TS exposure, WT mice were IP injected with or without CSP or CP once a day, 5 days/wk for the next 4 weeks while exposing the mice to TS for 20 weeks as we described earlier [7,15]. Lung sections were subjected to IHC using anti-IL-17A antibody (**i**). Whole lung homogenates were analyzed for IL-17A protein by Western blotting (**ii**). Total RNA extracted from lung homogenates was evaluated for IL-17A and IL-17RA mRNA expression by RT-qPCR (**iii**). (**B**) CSP inhibits IL-17A in the lungs of WT mice with BLM-LI. WT mice were exposed to saline or BLM, and mice with BLM-LI were treated with or without CSP or CP as we described earlier [2,6]. Lung sections were subjected to IHC using anti-IL-17A (**i**). Lung homogenates were analyzed for IL-17A protein by ELISA (**ii**), and total lung RNA isolated from WT or uPA^−/−^ mice with BLM-LI and treated with or without CSP or CP were analyzed for IL-17A mRNA by qPCR (**iii**).

CSP attenuates IL-17A levels in lungs of WT mice with TSE-LI and BLM-LI. IL-17A levels are enhanced in the lung tissues of WT mice during BLM-LI and TSE-LI, and IL-17A-deficient mice resist BLM- and TSE-induced p53-miR-34a feedback induction and AEC injury. In addition, CSP and its deletion fragments inhibit TSE-, BLM- and silica-induced AEC damage [6,7,10,13,16]. We therefore subjected lung sections from TSE-LI mice treated with CSP to IHC for IL-17A. Consistent with previous reports [46] and our findings above (Figure 1 and Figure 2), we found intense staining for IL-17A in the lung sections of WT mice with TSE-LI compared to control mice kept in ambient air. The staining of IL-17A in the lung tissues was markedly reduced in TSE-LI mice treated with CSP for four weeks, while lung sections of those exposed to CP of scrambled sequence still showed robust staining for IL-17A antigen (Figure 4(Ai)). This was further confirmed by immunoblotting of whole lung homogenates for IL-17A protein (Figure 4(Aii)). qPCR analysis of total lung RNA also confirmed significant inhibition of IL-17A and IL-17RA mRNA by treatment of TSE-LI mice with CSP (Figure 4(Aiii)). However, mice with TSE-LI left untreated or treated with CP showed elevated IL-17A protein and IL-17A and IL-17RA mRNA levels in their lung tissues. Since BLM-LI also augments IL-17A protein and mRNA in the lung tissues (Figure 1C), IL-17A-deficient mice resist BLM-LI and BLM-PF (Figure 2A), and CSP treatment attenuates early lung injury and fibrosis in WT mice [2,6], we next analyzed lung tissues of mice with BLM-LI treated with or without CSP or CP. As shown in Figure 4(Bi), BLM-LI showed increased IL-17A staining in lung tissues compared to the baseline IL-17A staining in control mice exposed to saline. This was markedly reduced in WT mice with BLM-LI and treated with CSP, while those exposed to CP still showed intense staining for IL-17A. Quantitation of IL-17A by ELISA of the lung tissues also showed a significant increase in IL-17A concentration in mice with BLM-LI (Figure 4(Bii)). This was reduced after treatment of WT BLM-LI mice with CSP but not CP. qPCR analysis of total RNA extracted from whole lung homogenates further proved that CSP treatment caused significant suppression of IL-17A mRNA, which was otherwise increased in BLM-LI mice left untreated or treated with CP (Figure 4(Biii)). Further analysis of lung tissues from uPA-deficient mice indicated that BLM-LI induced a significant expression of IL-17A mRNA compared to their baseline levels detected in corresponding control mice exposed to saline. Consistent with the inability of CSP to mitigate BLM-LI in uPA-deficient mice [6], CSP failed to significantly suppress IL-17A in uPA-deficient mice having BLM-LI.

### 2.5. IL-17A Triggers Apoptotic Signaling via p53 and PAI-1 in AECs

**Figure 5:**
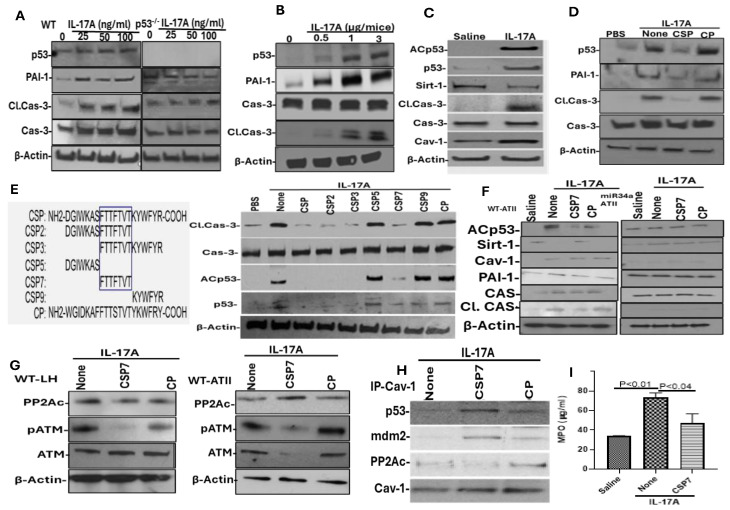
IL-17A induces p53, PAI-1, Cav1 and apoptosis, and CSP or CSP7 inhibits IL-17A-induced apoptosis in AECs. (**A**) AECs isolated from uninjured WT and p53^−/−^ mice were treated with IL-17A (0–100 ng/mL) for 24 h, and the lysates were immunoblotted for p53, PAI-1, Cl./Cas-3 and β-actin. (**B**) WT mice received saline or IL-17A intranasally (0–3 μg in 50 μL saline), as previously described [26]. AECs were isolated 24 h later, and lysates were immunoblotted for p53 and Cl./Cas-3 and β-actin. (**C**) WT mice were exposed to saline or IL-17A (1 μg), as described in (**B**). Lysates of AECs isolated 24 h after treatment with IL-17A were immunoblotted for ACp53, p53, Sirt1, Cav1 and apoptosis. (**D**) AECs isolated from WT mice were treated with PBS or IL-17A (100 ng/mL). Two hours later, IL-17A-stimulated cells were treated with or without CSP or CP (10 µM) and analyzed for p53, PAI-1 and apoptosis by Western blotting (WB). (**E**) AECs isolated from WT mice exposed to IL-17A (100 ng/mL) were treated with CSP, its overlapping deletion fragments, or control peptide (CP) for 24 h in vitro. The lysates were analyzed for ACp53, p53, PAI-1, Sirt1 and Cl. Cas-3 by WB. (**F**) miR-34a^fl/fl^ mice and miR-34acKO mice lacking miR-34a in AECs were exposed to IL-17A (1 μg) with or without CSP7 or CP. Control mice were exposed to saline. Lysates from AECs extracted from these mice were analyzed for p53, PAI-1, Sirt1, Cav1 and apoptosis by WB. (**G**) Lung homogenates or lysates of AECs extracted from WT mice exposed to IL-17A with or without CSP7 or CP were subjected to WB for PP2Ac, pATM and ATM kinase. (**H**) Lung homogenates of IL-17A-exposed mice treated with or without CSP7 or CP were immunoprecipitated (IP) using anti-Cav1 antibody and immunoblotted for p53, mdm2, and PP2Ac. (**I**) Lung homogenates of WT mice exposed to saline or IL-17A with or without CSP7 were analyzed for MPO activity.

Because CSP mitigates IL-17A in mice with BLM-LI and TSE-LI, we isolated AECs from WT mice and treated them with varying concentrations (0–100 ng/mL) of recombinant IL-17A to determine whether IL-17A alone can induce AEC injury through the induction of p53 and downstream upregulation of PAI-1. As shown in Figure 5A, treatment of AECs with IL-17A caused a dose-dependent increase in cleaved caspase-3, suggesting apoptosis with parallel induction of p53 and PAI-1. However, treatment of p53^−/−^ AECs with IL-17A failed to induce apoptosis or PAI-1 expression, suggesting that p53-induced PAI-1 plays a vital role in AEC apoptosis. To further confirm the above findings in vivo, we next treated WT mice with escalating doses (0–3 μg/20 g mice) of recombinant IL-17A, isolated AECs from these mice, and analyzed them for activated caspase-3 as a surrogate marker of apoptosis as well as for the expression of Cav1, p53 and PAI-1 to assess whether Cav1-dependent induction of p53 and downstream PAI-1 contributes to AEC apoptosis. Results showed that the expressions of p53, PAI-1, Cav1 and apoptosis were increased in a dose-dependent manner in AECs of WT mice. Maximum expression of these proteins was observed at a concentration of 1–3 μg/20g body weight (Figure 5B). Consistent with an increase in p53, treatment of mice with IL-17A also caused marked induction of ACp53 and inhibition of Sirt1, which was otherwise elevated in naïve mice (Figure 5C), suggesting that increased p53-miR-34a feedback induction and miR-34a-mediated inhibition of Sirt1 contribute to activation of p53 and AEC injury in mice exposed to exogenous IL-17A. IL-17A induces apoptosis by inducing dose-dependent expression of p53 and its downstream mediator (PAI-1) in AECs treated in vitro or in WT mice treated in vivo. Further, CSP treatment improves AEC viability and inhibits IL-17A in WT mice with BLM-LI or TSE-LI. To directly test whether CSP inhibits IL-17A-induced AEC apoptosis by blocking p53-uPA system crosstalk, we treated AECs with IL-17A in the presence or absence of CSP or CP. As shown in Figure 5D, IL-17A-induced apoptosis in AECs was indicated by a marked increase in cleaved caspase-3 with parallel induction of p53 and PAI-1 expression compared to control cells treated with PBS alone. However, treatment of AECs exposed to IL-17A with CSP, but not CP, attenuated apoptosis, as depicted by the suppression of IL-17A-mediated induction of active caspase-3, p53 and PAI-1. To identify the minimal active component of CSP contributing to the anti-apoptotic response against IL-17A in AECs, we made a series of overlapping deletions of CSP (Figure 5E) and treated IL-17A-exposed AECs with full-length CSP or its various overlapping deletion fragments. We found that treatment of IL-17A-titrated AECs with fragments of CSP (CSP2, CSP3 and CSP7), which contained the amino acids FTTFTVT, inhibited p53 and caspase-3 activation. However, CSP5 and CSP9, which did not include this sequence, had no effect.

IL-17A treatment inhibits Sirt1 expression and augments ACp53 in AECs, and microRNA-34a blocks Sirt1 mRNA translation, leading to the accumulation of ACp53. We next generated tamoxifen-inducible conditional knockout (miR-34acKO) mice lacking miR-34a expression in AECs by cross-breeding miR-34afl/fl mice with SP-CCre/mTmG mice, as we had reported elsewhere [2]. We next exposed miR-34afl/fl and miR-34acKO mice to IL-17A with or without IP injection of CSP7 or CP. We isolated AECs from miR-34afl/fl and miR-34acKO mice and tested for changes in Sirt1, Cav1, p53 and PAI-1 expression and apoptosis. IL-17A induced Cav1, p53, ACp53, PAI-1 and cleaved caspase-3 while suppressing the expression of Sirt1 compared to saline-treated control mice, indicating AEC injury. However, treatment of WT or miR-34afl/fl mice exposed to IL-17A with CSP7 reduced p53, PAI-1 expression, and apoptosis while restoring baseline Sirt1 without affecting Cav1 in AECs (Figure 5F), suggesting attenuation of IL-17A-induced lung injury. Consistent with the lack of TSE- or BLM-induced miR-34a induction or lung injury in miR-34acKO mice [2,15], IL-17A failed to induce p53 or PAI-1 expression or inhibit Sirt1 in AECs of miR-34acKO mice. These mice also resisted AEC apoptosis. Further, CSP7 had a minimal effect in miR-34acKO mice exposed to exogenous IL-17A. Thus, our findings implicate miR-34a in TSE-LI and further show that, through the inhibition of miR-34a, CSP7 reverses TSE- or IL-17A-induced p53, downstream PAI-1, and apoptosis in AECs.

Besides induction of miR-34a-mediated ACp53, P15Sp53 by ATM kinase prevents mdm2-mediated ubiquitination of p53, leading to its stabilization during BLM-LI and TSE-LI. CSP or CSP7 inhibits sequestration of the catalytic subunit of protein phosphatase (PP) 2A (PP2Ac), resulting in dephosphorylation and inactivation of ATM kinase, inhibition of serine phosphorylation of ATM (pATM) kinase, attenuation of pATM kinase-mediated P15Sp53, and degradation of p53 by mdm2 [2,10]. To determine whether Cav1 induced by IL-17A corresponds to increased P15Sp53, we screened for differential expression of ATM and pATM using total lung homogenates and AECs isolated from WT mice treated with IL-17A. We found that IL-17A induced pATM kinase in lung tissues and isolated AECs from these mice (Figure 5G), which were inhibited in mice exposed to IL-17A and treated with CSP7. Since Cav1 can sequester PP2Ac through its Cav1 scaffolding domain, we immunoprecipitated the total lung homogenate of WT mice treated with IL-17A using an anti-Cav1 antibody and immunoblotted them for PP2Ac, p53 and mdm2 to confirm whether CSP7 blocks Cav1-mediated sequestration of PP2Ac or mdm2. As shown in Figure 5H, mice treated with IL-17A augmented the Cav1 and PP2Ac interaction, and treatment with CSP7 inhibited the Cav1-PP2Ac binding when compared to treatment with CP. Mice treated with CSP7 showed reduced Cav1 interaction with PP2Ac, suggesting that CSP7 inhibits Cav1-mediated sequestration of PP2Ac, leading to inactivation of ATM kinase by PP2Ac, loss of P15Sp53, and degradation of p53 by mdm2. Consistent with TSE-LI or BLM-LI, we found that the lung tissues of WT mice exposed to IL-17A showed increased MPO activity compared to saline-treated control mice (Figure 5I). This was significantly reduced in the lung tissues of mice subjected to post-IL-17A CSP7 treatment.

### 2.6. The Axis of IL-17A/IL-17RA in Progressive PF

**Figure 6:**
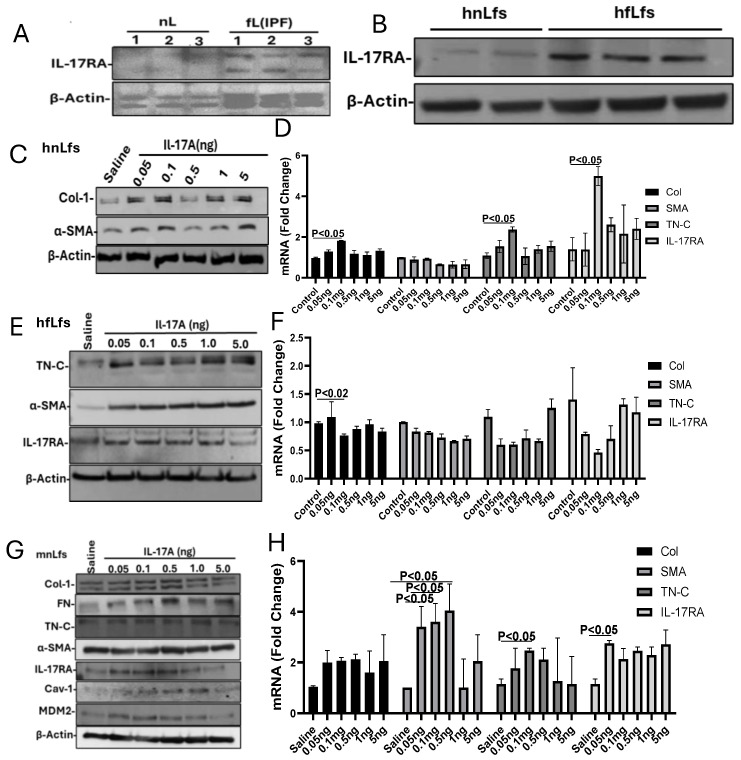
Role of IL-17A and IL-17RA on fLfs differentiation. Increased IL-17RA expression in IPF lung tissues (**A**) and in fLfs (**B**) derived from IPF tissues. Dose-dependent induction of IL-17RA and profibrogenic markers by IL-17A. Human nLfs treated with varying (0–5 ng/mL) concentrations of IL-17A were analyzed for IL-17RA and profibrogenic marker (**C**) proteins and (**D**) their mRNAs. Induction of IL-17RA and profibrogenic marker proteins (**E**) and their mRNAs (**F**) in fLfs from human IPF lungs by IL-17A. IL-17A induces IL-17RA and profibrogenic markers in mouse nLfs. nLfs isolated from naïve mice were treated with IL-17A (0–5 ng/mL), and Lfs lysates were analyzed for IL-17RA, Cav1, mdm2 and profibrogenic marker proteins (**G**) and some of their mRNAs (**H**).

Besides AEC injury and loss of alveolar epithelial regeneration, post-lung injury, differentiation and expansion play a crucial role in progressive PF. Since the biological activity of IL-17A depends upon its interaction with its receptor, IL-17RA, we tested lung homogenates of IPF tissues for IL-17RA expression and found a marked increase in IL-17RA in IPF lung tissues compared to the IL-17RA levels in three non-IPF control lung tissues tested (Figure 6A). Consistent with elevated expression of IL-17RA in IPF tissues, we found f LFs extracted from IPF tissues also showed increased steady-state expression of IL-17RA (Figure 6B). To determine whether IL-17A promotes f LF activation and differentiation, we treated n LFs from non-IPF donor lungs and f LFs from patients with IPF with 0–5 ng/mL of recombinant IL-17A and tested profibrogenic markers such as Col1 and α-SMA by Western blotting. We found that exogenous IL-17A caused a dose-dependent increase in Col1 and α-SMA expression in n LFs (Figure 6C), suggesting that IL-17A promotes fLf differentiation. This was further confirmed by a dose-dependent increase in Col1, FN and TN-C mRNA expression in human nLfs (Figure 6D). Since IL-17A and IL-17RA levels are elevated in IPF lungs, we treated fLfs isolated from IPF lungs with different doses of IL-17A and found that IL-17A likewise augments the expression of profibrogenic proteins such as α-SMA and TN-C (Figure 6E) and their mRNAs (Figure 6F). Consistent with the increase in profibrogenic marker expression, IL-17A treatment also caused a dose-dependent increase in IL-17RA expression in both human nLfs and fLfs from IPF lungs. Western blotting (Figure 6G) and qPCR analysis (Figure 6H) of nLfs isolated from naïve mice treated with IL-17A revealed a dose-dependent increase in profibrogenic marker proteins and their transcripts, accompanied by a parallel upregulation of IL-17RA protein and mRNA. These results collectively indicate that elevated IL-17A/IL-17RA signaling promotes PF progression by targeting multiple cell types, including AECs, LFs, and likely additional cell populations.

**Figure 7:**
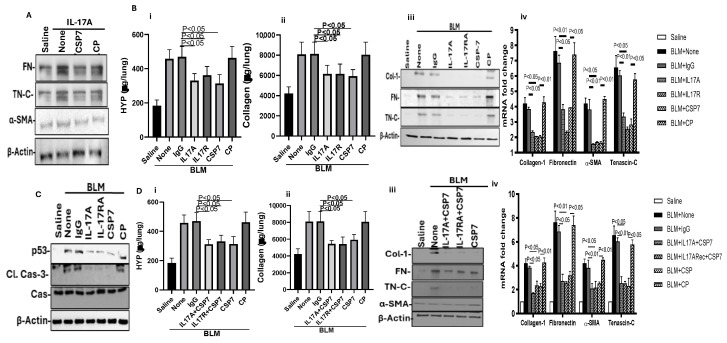
Targeting IL-17A-mediated differentiation using CSP7 and anti-IL-17A and anti-IL-17RA antibodies attenuates PF. (**A**) nLfs isolated from naïve mice were exposed to IL-17A, and 24 h later, IL-17A-stimulated cells were treated with or without CSP7 or CP for an additional 24 h. Lf lysates were later immunoblotted for IL-17A-induced changes in profibrogenic marker proteins. (**B**) WT mice having BLM-PF were treated with or without control IgG, anti-IL-17A antibody, anti-IL-17RA antibody or CSP7 alone. Saline-treated mice were used as a control for comparison. Whole lung homogenates were analyzed for total HYP content (**i**) and S Col (**ii**) contents and profibrogenic marker proteins by Western blotting (WB) (**iii**). Total lung RNA was analyzed for Col1, FN, α-SMA and TN-C mRNAs (**iv**). (**C**) AECs isolated from BLM-PF mice treated as in (**B**) were analyzed for p53 and Cl. Caspase-3 and total caspase-3 by WB. (**D**) WT mice exposed to BLM were treated with or without CSP7 alone or in combination with anti-IL-17A antibody or anti-IL-17RA antibody. Twenty-one days after initiation of BLM-LI, mice were euthanized, and whole lung homogenates were analyzed for total HYP content (**i**) and S Col (**ii**) contents, profibrogenic marker proteins by WB (**iii**) and their mRNAs by qPCR (**iv**).

IL-17A and its receptor, IL-17RA, are elevated in fibrotic lung tissues, and the IL-17A/IL-17RA signaling axis concurrently promotes AEC apoptosis and senescence, leading to reduced alveolar epithelial repair while promoting activation and expansion of fLfs. Therefore, we next sought to understand whether IL-17A can induce activation of nLfs and if CSP7 inhibits IL-17A-induced activation of nLfs. As shown in Figure 7A, treatment of mouse nLfs with IL-17A induced profibrogenic marker proteins such as FN, TN-C and α-SMA, suggesting fLfs differentiation. This was reduced following treatment of IL-17A-stimulated cells with CSP7 but not the control peptide, CP. Since IL-17A and IL-17RA are increased in IPF lung tissues, and CSP or CSP7 inhibits AEC death and PF, we treated mice harboring PF with anti-IL-17A or anti-IL-17RA antibody or CSP7 alone daily for 7 days starting on day 14 through to day 21 post-BLM. We found that treatment of mice with BLM-PF with either anti-IL-17A or anti-IL-17RA antibody or CSP7 alone caused a significant reduction in BLM-induced total lung HYP (Figure 7(Bi)) and sCol (Figure 7(Bii)) content compared to BLM-PF mice left untreated. However, analysis of whole lung homogenates for total HYP and sCol content in BLM-PF mice exposed to control IgG or CP used as comparable controls in the study still showed elevated levels. Immunoblotting of lung homogenates demonstrated elevated expression of Col1, FN, α-SMA and TN-C in control WT mice exposed to BLM-PF and left untreated or treated with IgG or CP. This was markedly reduced in BLM-PF mice treated with anti-IL-17A antibody, anti-IL-17RA antibody, or CSP7 (Figure 7(Biii)). Analysis of total RNA extracted from the whole lung tissues for Col1, FN, α-SMA and TN-C mRNA further confirmed a nearly similar antifibrogenic response (Figure 7(Biv)). Since increased IL-17A/IL-17RA and AEC apoptosis promote BLM-PF and IL-17A-deficient mice resist BLM-PF, we tested AECs isolated from BLM-PF mice exposed to CSP7, anti-IL-17A or anti-IL-17RA antibody for apoptosis. As shown in Figure 7C, AECs of BLM-PF mice treated with CSP7, anti-IL-17A or anti-IL-17RA antibody reduced p53 and Cl. caspase-3, suggesting protection of AECs. Combination therapy of anti-IL-17A or anti-IL-17RA antibody with CSP7 did not further reduce total HYP (Figure 7(Di)) and sCol (Figure 7(Dii)) content or profibrogenic marker proteins (Figure 7(Diii)) or their mRNAs (Figure 7(Div)), indicating overlapping responses.

## 3. Discussion

Both chronic restrictive and obstructive lung diseases are usually progressive and irreversible and are often associated with age and abnormal inflammatory responses of the lungs. This is due to long-term exposure to TS, particulates or noxious gases, which can induce reactive oxygen species and DNA damage [50,51,52,53,54,55]. Lung parenchymal exposure to particulates and DNA-damaging agents such as TS, silica, TR, BLM and several other chemotherapeutic agents promotes the influx of inflammatory cells, including CD4^+^ and CD8^+^ T cells, neutrophils and macrophages, causing chronic inflammation. AEC viability and renewal play a vital role in parenchymal regeneration and tissue homeostasis by regulating proliferation and differentiation following lung injury. CD4^+^ and CD8^+^ T cells may induce AEC apoptosis through the release of IL-17A and a number of cytokines and mediators [56]. IL-17A triggers the production of numerous chemokines, which in turn promote PMN and macrophage influx and thereby facilitate crosstalk between the adaptive and innate immune systems [20,21]. IL-17A expression has also been linked to reduced steroid responsiveness [57,58], which suggests that IL-17A and related cytokines may play a role in the development of steroid-resistant chronic lung diseases, including IPF and COPD. Elevated IL-17A levels in the alveoli promote a COPD-like phenotype and exacerbate BLM-PF in mice [30], and it is also an important mediator of chronic ILDs and connective tissue disorders [59]. IL-17A is markedly increased in the lungs and sputum of patients with COPD and progressive PF such as IPF [30,59]. IL-17A mediates cellular signaling by binding to IL-17RA, and the IL-17A/IL-17RA signaling axis has been implicated in the pathogenesis of various diseases, including chronic lung disorders [20,21,22]. Therefore, to examine the potential significance of elevated IL-17A in lung injury and lung remodeling, we exposed IL-17A-deficient mice to TS and BLM at different times, and AECs isolated from these mice were analyzed for changes in telomere shortening, shelterin complex binding proteins, p53 and PAI-1, as well as profibrogenic markers, to assess cell viability and PF. Mice exposed to IL-17A and treated with CSP or CSP7 were evaluated for pulmonary inflammatory cytokines and chemokines, p53, PAI-1, AEC viability, and pro-fibrotic marker proteins. These findings were further validated using primary AECs treated with IL-17A, as well as lung tissues and AECs isolated from WT and p53^−/−^ or PAI-1-/- mice exposed to TS or IL-17A. Additionally, lung tissues, AECs, lung homogenates, lung lavage fluids, and RNA samples were assessed for alterations in IL-17A, IL-17RA, p53, PAI-1, and activated caspase-3 using IHC, immunoblotting, and qPCR assays.

Consistent with findings in BLM-LI and TSE-LI models, IL-17A induced both total and ACp53 apoptosis while suppressing Sirt1 in AECs. This suggests that inhibition of Sirt1-mediated deacetylation of p53 contributes to the accumulation of ACp53 and p53, leading to AEC apoptosis. Treatment with either CSP or CSP7 inhibited IL-17A-induced apoptosis, a process that involves Sirt1-mediated deacetylation and subsequent degradation of p53 in AECs. To confirm whether CSP7 mimics the effect of CSP in vivo, we treated WT TSE-LI mice with or without CSP7. Isolated AECs were tested for IL-17RA, PAI-1, ACp53, P15Sp53, apoptosis and senescence. All were increased in TSE-LI mice, whereas they were markedly reduced following treatment with CSP7. We previously found that miR-34a and p53 levels are increased in AECs after TSE-LI and BLM-LI, which is significantly inhibited by CSP in WT mice [2]. In addition, we also reported that increased miR-34a augmented p53 and apoptosis in AECs without TSE-LI or BLM-LI, while inhibition of miR-34a using miR-34a antisense reduced BLM- or TSE-induced p53 and apoptosis. Results of both in vitro and in vivo experiments support an intricate link between miR-34a, ACp53, p53 and p53-mediated induction of PAI-1 and AEC apoptosis during TSE-LI and BLM-LI.

CSP significantly inhibited TSE-induced IL-17A and IL-17RA levels. Further, this process involves the induction of p53 and upregulation of PAI-1 in AECs. To confirm this, we treated primary AECs with varying concentrations of IL-17A. Consistent with TSE-induced AEC injury, IL-17A treatment alone led to increased expression of both p53 and PAI-1, as well as apoptosis in AECs. We also found that IL-17A concurrently induced ACp53 and P15Sp53, indicating that posttranslational stabilization of p53 contributes to its increased steady-state expression. The IL-17A-induced upregulation of p53 in AECs was associated with a concurrent increase in Cav1 and miR-34a-mediated suppression of Sirt1. Consistent with resistance to TSE-LI by p53 deficiency, IL-17A failed to induce PAI-1 or apoptosis in AECs lacking p53 expression. We next exposed the WT mice to recombinant IL-17A, and AECs were isolated 24 h later. The AEC lysates were tested for induction of ACp53, p53, Cav1 and apoptosis. Consistent with TSE-LI or BLM-LI in WT mice, exogenous IL-17A markedly increased total and ACp53 levels, apoptosis, and Cav1 expression in AECs. In contrast, IL-17A failed to induce PAI-1 or apoptosis in p53-deficient mice. These findings indicate that IL-17A recapitulates the effects of TSE-LI and BLM-LI and highlight the pivotal role of IL-17A in p53-mediated AEC apoptosis and lung injury.

Besides miR-34a-mediated acetylation, increased interaction of acyltransferase p300 with P15Sp53 increases ACp53. These changes reduce binding and degradation of ACp53 and P15Sp53 by mdm2. TSE-LI and BLM-LI also induced Cav1 and reduced Sirt1 in AECs, suggesting that inhibition of Sirt1-mediated deacetylation contributes to induction of ACp53, PAI-1 and apoptosis. In addition, treatment of WT TSE-LI or BLM-LI mice with CSP or CSP7 reduced ACp53, P15Sp53 and p53 levels and restored baseline Sirt1 expression without inhibiting Cav1 in AECs. We further found that both TSE and IL-17A exposure augmented Cav1 expression and Cav1 binding with PP2Ac, which in turn sequestered PP2Ac from the cytoplasm to the caveoli. This led to the activation of ATM kinase through phosphorylation of its serine 1981 and downstream ATM kinase-mediated P15Sp53 and ACp53, leading to stabilization of p53. This was completely reversed by treatment of TSE-LI mice with CSP, which inhibited Cav1 binding with PP2Ac.

Increased p53 and apoptosis in AECs of TSE-LI or IL-17A-treated mice provided evidence that IL-17A contributes to lung injury. To confirm that increased IL-17A contributes to the induction of p53 and PAI-1 and apoptosis in AECs during TSE-LI, we exposed IL-17A-deficient mice to 20 weeks of TS. AECs were tested for ACp53, p53 and Sirt1 expression, and apoptosis. The responses were compared with changes in AECs isolated from WT mice exposed to 20 weeks of TSE or air. We observed marked induction of ACp53 and p53 levels and apoptosis with parallel suppression of Sirt1 in WT TSE-LI mice. However, TSE failed to induce ACp53 or p53, PAI-1 and apoptosis or inhibit Sirt1 in AECs, indicating resistance by mice lacking IL-17A expression. To confirm mitigation of TSE-LI by CSP or CSP7 involves suppression of IL-17A-induced p53, PAI-1 and apoptosis, we next treated AECs from WT mice with IL-17A (100 ng/mL) with or without CSP, CSP7 or CP. The AEC lysates were tested for p53 and downstream induction of PAI-1 and apoptosis. Both CSP and CSP7 inhibited IL-17A-induced p53, PAI-1 expression, and apoptosis in AECs. Similarly, treatment of WT mice exposed to IL-17A with either CSP or CSP7 reduced AEC p53, PAI-1 and apoptosis in vivo. Consistent with the marked elevation of IL-17A levels in the lungs of patients with COPD [21,40], WT mice with TSE-LI showed a significant increase in both IL-17A protein and mRNA. Unlike WT mice, mice deficient in IL-17A resisted TSE-LI, suggesting that IL-17A plays a pivotal role in lung injury. We found that IL-17A induces p53 and PAI-1 expression and apoptosis in a dose-dependent manner in WT AECs. However, p53-deficient AECs treated with IL-17A failed to induce PAI-1 or apoptosis, demonstrating that the process is mediated through the induction of endogenous p53. To confirm this occurs in vivo, we treated WT mice with 0–3 μg of IL-17A per 20 g body weight for 24 h and tested isolated AECs from these mice for p53, PAI-1 and activation of caspase-3 to assess apoptosis. We observed maximum induction of p53 and PAI-1 and apoptosis with 1.5 and 3 mg of IL-17A. These changes were associated with dose-dependent induction of ACp53 and Cav1, with concurrent suppression of Sirt1 histone deacetylase, suggesting that Sirt1-mediated deacetylation controls the p53 steady-state levels in AECs. Further, activation of p53 through P15Sp53 potentiates its acetylation and contributes to its steady-state increase by preventing mdm2-mediated degradation.

p53 induces miR-34a transcription, while miR-34a induces p53 protein stabilization by blocking Sirt1-mediated deacetylation. To directly confirm the importance of increased miR-34a in IL-17A-induced p53 expression and lung injury, we generated tamoxifen-inducible miR-34a conditional knockout (miR-34acKO) mice lacking miR-34a in AECs [2]. These mice were treated with IL-17A. WT mice treated with IL-17A served as controls. The responses were compared with miR-34acKO mice exposed to 20 weeks of TS. We found that AECs from miR-34acKO mice resisted both IL-17A and TSE-LI. However, WT mice were responsive to both IL-17A- and TSE-induced AEC injury, which was reversed after treatment of WT mice with CSP7. Further, analyses of isolated AEC lysates revealed that IL-17A or TSE failed to induce ACp53, PAI-1 and apoptosis or suppress Sirt-1, indicating resistance of miR-34acKO mice to lung injury.

Our findings establish IL-17A as a pivotal driver of AEC injury and fibrotic remodeling through a p53-dependent pathway, with direct implications in chronic lung diseases such as IPF and COPD. IL-17A signaling via IL-17RA stabilizes p53 through phosphorylation and acetylation while simultaneously inducing miR-34a transcription, which suppresses Sirt1-mediated deacetylation, forming a feed-forward loop that sustains p53 activation. Activated p53 upregulates PAI-1 and suppresses uPA/uPAR, disrupting the fibrinolytic balance, reducing AEC viability by promoting epithelial senescence and apoptosis, and impairing alveolar regeneration and repair. These epithelial alterations create a profibrotic microenvironment, driving fibroblast activation, myofibroblast differentiation, and excessive ECM deposition, culminating in lung remodeling and progressive fibrosis. Clinically, elevated IL-17A levels in COPD and IPF patients relate to increased epithelial cell apoptosis, persistent inflammation, and steroid resistance, suggesting that uncontrolled IL-17A signaling contributes to disease progression and limits therapeutic responsiveness. Importantly, our results show that CSP and CSP7 effectively disrupt the IL-17A–p53–PAI-1 axis, restore Sirt1 activity, reduce p53/PAI-1 expression, and prevent AEC apoptosis, indicating that targeting this pathway could be a viable strategy to mitigate chronic lung injury and consequent fibrotic remodeling in patients with inflammatory or fibrotic lung diseases.

**Figure 8:**
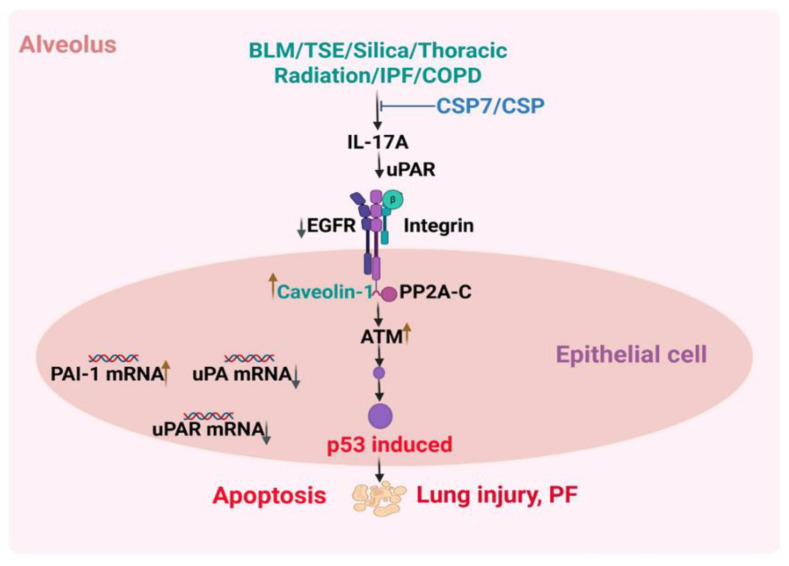
Role of IL-17A, p53 and the uPA fibrinolytic system in the regulation of BLM-LI and TSE-LI. IL-17A is increased during BLM-LI, TSE-LI or other lung injuries. IL-17A induces p53 in AECs. p53 inhibits uPA and uPAR expression while inducing PAI-1 expression. PAI-1 inhibits the enzymatic activity of uPA and promotes uPA-uPAR internalization and degradation. Inhibition in uPA and uPAR expression and induction of PAI-1 expression are correlated with increased p53 and miR-34a expression in AECs. Together, these changes induce AEC apoptosis and PF. CSP or CSP7 reduces p53 and PAI-1 and enhances uPA expression in AECs. CSP or CSP7 blocks p53 via cell surface signaling that involves Cav1, uPAR, β1-integrin and Src kinase. AEC apoptosis and PF can be reversed by interruption of this pathway via CSP or CSP7.

In conclusion, mouse models of lung injury induced by various agents showed elevated IL-17A production and its involvement in AEC apoptosis and lung injury, suggesting the essential role of IL-17A in chronic lung injury (Figure 8). The process involves miR-34a-p53 feed-forward induction and downstream PAI-1 expression. Our results also show that IL-17A augments Cav1, p53, p53 downstream targets, including PAI-1 and miR-34a, in AECs, thereby promoting senescence and apoptosis, which are central to the pathogenesis of diverse types of lung injuries and fibrotic remodeling. IL-17A-, p53- and PAI-1-deficient mice resist BLM-, silica- and TSE-induced AEC senescence or apoptosis, as well as lung injury and remodeling [2,3,6,7,12,13,14,15,16]. Similarly, mice lacking p53 or miR-34a expression in AECs also resist BLM-LI and TSE-LI. Our results further show that inhibition of IL-17A-mediated induction of p53 and PAI-1 crosstalk in AECs by CSP (a 20-amino-acid peptide derived from the scaffolding domain of caveolin-1) or CSP7 (a 7-amino-acid deletion peptide derived from CSP) mitigates apoptosis and prevents subsequent development of PF.

## 4. Materials and Methods

### 4.1. IL-17A Expression in Human Lung Tissues of Patients with IPF and COPD

All studies involving de-identified human lung tissue specimens derived from surgical biopsies or harvested at the time of lung transplantation or autopsy of patients with IPF or control donors without known lung disease were approved by the institutions involved, including the University of Texas Health Science Center at Tyler, TX, USA. Lung tissues were provided by the Lung Tissue Research Consortium or obtained from the Department of Thoracic Medicine and Surgery, Temple University, Philadelphia, PA, USA. The clinical diagnosis of patients with IPF and COPD was established by histology, physical examination, CT and pulmonary function tests. The pathological diagnosis of IPF was usual interstitial pneumonia (UIP) based on the presence of restrictive lung disease in association with a compatible clinical presentation and lung pathology. COPD tissues were obtained from patients classified as GOLD4 by the GOLD classification. The lung sections from patients with IPF, COPD, and control donors were subjected to immunohistochemical (IHC) analysis, and lung homogenates were subjected to immunoblotting and qPCR analyses to assess changes in IL-17A.

### 4.2. Mice

Wild-type (WT) C57BL/6 mice and breeding pairs of p53-/-, PAI-1-/- and uPA-/- mice were purchased from Jackson Laboratory (Bar Harbor, ME, USA). Breeding pairs of IL-17A-/- mice were obtained from the University of Tokyo, Tokyo, Japan. PAI-1-/-, uPA-/- and IL-17A-/- mice of C57BL/6 background were bred in our facility. WT and p53-/-, PAI-1-/-, uPA-/- and IL-17A-/- male and female mice were used for the experiments. All breeding, care, and experimental procedures followed protocols approved by the Institutional Animal Care and Use Committee (IACUC, Memphis, TN, USA) to ensure ethical treatment and compliance with regulatory standards.

### 4.3. Development of Mouse Models of Chronic Tobacco Smoke Exposure (TSE) Induced Lung Injury (TSE-LI) and Testing Salutary Effects of CSP and CSP7

To investigate the effect of TSE-LI, WT, and p53-/-, PAI-1-/-, uPA-/- and IL-17A-/- mice were exposed to passive TS from 40 research cigarettes over a 4 h period, 5 days/week for 20 weeks (~90 mg/m^3^ total solid particulates), using a mechanical smoking chamber (Teague Enterprises, Davis, CA, USA) as we reported in our publications [3,7,15]. Mice kept in ambient air were used as controls. After sixteen weeks of daily exposure to TS, the mice were treated with 1.5 mg/kg body weight of CSP, CSP7, or a control peptide of scrambled sequence (CP) by intraperitoneal (IP) injection daily, 5 days/week for four weeks. Daily TS exposure of 4 h was continued during the peptide treatment period. After 20 weeks of passive TS exposure, mice were euthanized, and lung lavage fluids, lung tissues, and isolated AECs from the extracted lung tissues were collected for further analysis.

### 4.4. Development of Mouse Models of BLM-LI and BLM-PF and Testing the Beneficial Effect of CSP or CSP7

WT or IL-17A-, uPAR-, PAI-1- and p53-deficient mice were exposed to a single dose (1X) of BLM in the presence of CSP or CSP7 or CP (1.5 mg/kg body weight) as we described earlier [2,4,12,13,16]. Mice were either euthanized 1-3 days after BLM exposure to assess changes in early lung injury or 21 days later to evaluate changes in BLM-PF as we described in our recent publications [2,5,13,14]. Control mice were exposed to vehicle (normal saline). Mice with BLM-LI were treated with or without CSP, CSP7 or CP one day after exposure to BLM, whereas BLM-PF mice were treated with peptides (CSP7 or CP) or anti-IL-17A antibody, anti-IL-17RA antibody, anti-IL-17A antibody + CSP7 or anti-IL-17RA antibody + CSP7 14 days after initiation of BLM injury, as we described elsewhere [12,13,14]. At the end of the experiment (days 1, 2, 3 and 21 post-BLM), the mice were euthanized, and lung lavage fluids, lung tissues, and isolated AECs from the lung tissues were collected for further analysis.

### 4.5. Development of Mouse Models of Silica-Induced Lung Injury and PF (Silicosis) and CSP or CSP7 Treatment

WT mice were exposed to 1X of crystalline silica (MIN-U-SIL-5 @ 1.5 mg/100 μL) or vehicle (saline) as we described previously [10,16,60]. Upon completion of the treatment period, the mice were euthanized, and their lungs were collected for the preparation of homogenates and histopathological tissue sections and for the isolation of AECs for further ex vivo analysis.

### 4.6. Development of Mouse Models of Thoracic Radiation (TR)-Induced Lung Injury (TR-LI) and TR-Induced PF (TR-PF)

WT mice received doses of 10 or 20 gray units (Gy) of thoracic radiation as mentioned previously [11,61], while control mice were not exposed to any treatment. After a period of 28 days, all mice, including both naïve and those subjected to TR, were euthanized. Lung tissues and AECs were extracted for subsequent analysis.

### 4.7. Mouse Model of IL-17A-Induced Lung Injury (IL-17A-LI)

WT mice received different doses of IL-17A (ranging from 0 to 3 μg/20 g mice) by intranasal instillation in a total volume of 50 μL saline, as previously detailed [62]. Control mice were treated with the vehicle alone (50 μL saline). All mice were euthanized, and isolated AECs were further analyzed following the previously established protocol [15,16,63] for further analysis.

### 4.8. Isolation of AECs from Mouse Lungs

AECs isolated from WT mice and mice lacking PAI-1, uPA and IL-17A expression, as described elsewhere [3,5,50] were maintained in poly-L-lysine-coated plates containing growth-supplemented AEC culture medium (AEpiCM, ScienCell, Carlsbad, CA, USA) at 37 °C in an incubator supplied with 5% CO_2_ until use.

### 4.9. Treatment of AECs with IL-17A with or Without CSP or CSP7 In Vitro

AECs isolated from uninjured WT and p53-deficient mice were treated with IL-17A (0–100 ng/mL) in the presence or absence of CSP or CSP7 for 24 h, and the AEC lysates were immunoblotted for changes in p53 and PAI-1 expression, cleaved caspase-3, caspase-3 and β-actin. AECs with IL-17A-induced injury treated with CSP or CSP7 were analyzed for p53 and PAI-1 expression and AEC viability. Lung tissues, AECs, lung homogenates, lung lavage fluids, and RNA were evaluated for changes in IL-17A, IL-17RA, p53, PAI-1, and activated caspase-3 using IHC, immunoblotting, and qPCR assays.

### 4.10. Isolation, Culturing, and Treatment of LFs with IL-17A and CSP7

Mouse normal LFs (mnLfs) were isolated from naïve mice following the protocol described elsewhere [14]. Human “normal” Lfs (hnLfs) and IPF Lfs (hfLfs) purchased from Lonza were cultured in FGF2, insulin and serum-supplemented fibroblast growth medium, as described earlier [14]. mnLfs, hnLfs and hfLfs were treated with varying doses of IL-17A in the presence or absence of CSP7 or CP. LF lysates were subjected to Western blotting and qPCR analyses.

### 4.11. Protein Analysis by Western Blotting

Mouse AECs and mouse LFs (mnLfs) or human LFs (hnLfs and hfLfs) were lysed in radioimmunoprecipitation assay (RIPA) buffer (Pierce) containing protease inhibitors (Roche, Germany) and phosphatase inhibitors (Sigma-Aldrich, St. Louis, MI, USA). Whole lung homogenates and isolated cell (AEC, hnLfs, hfLfs and mnLfs) lysates were subjected to immunoblotting using p53, serine 15 phosphorylated p53 (P15Sp53), lysine 379 acetylated p53 (ACp53), caspase-3, cleaved caspase-3, PAI-1, IL-17A, IL-17RA, Cav1, catalytic subunit of protein phosphatase 2A (PP2Ac), ataxia-telangiectasia mutated (ATM), serine 1981 phosphorylated ATM (pATM), mouse double minute 2 (MDM2), sirtuin 1 (Sirt1), collagen1 α1 (Col1), fibronectin (FN), α-smooth muscle actin (α-SMA), tenascin-C (TN-C) and β-actin antibodies at 1:500–1:1000 dilution (Table 1) and donkey anti-rabbit/mouse/goat horseradish peroxidase-conjugated secondary antibody at 1:2000 dilution. The protein bands were visualized using an enhanced chemiluminescence (ECL, Thermo, Rockford, IL, USA) detection as we described previously [7,14,16].

### 4.12. Detection of Telomerase Activity

AECs were isolated from IL-17A^−/−^ mice kept in ambient air or exposed to 20 weeks of TS with or without CSP7 or CP treatment. The relative telomere length was analyzed by qPCR analysis of the genomic DNA using primer sequences listed in our recent publication [15].

### 4.13. mRNA Quantitation by Real-Time qPCR

Aliquots of total RNA isolated from AECs and LFs using TRI reagent were subjected to reverse transcription and quantitative real-time PCR using gene-specific primers purchased from Bio-Rad and Sigma as follows: p53 #qMmu CID0006264, FN1 #qMmuCED0045687, Col-1 #qMmuCED0044222, TNC #qHsaCED0057376, α-SMA #qMmuCED0027505, Gapdh #qMmuCED0027497, and (Table 2), as described earlier [7,16].

### 4.14. IHC Analysis of Lung Sections

Formalin-fixed and paraffin-embedded lung tissue sections (5.0 µm) were subjected to immunostaining using primary antibodies diluted in blocking buffer and further processed as we previously described [2,7].

### 4.15. Statistical Analysis

Each experiment was replicated (*n* = 4–5). The significance of the differences between experimental groups was evaluated by one-way ANOVA followed by Tukey’s post-hoc test using GraphPad Prism 4.0 software, as described earlier [14].

## Figures and Tables

**Table 1:** List of Antibodies.

	Antibody	Source	Catalog No	Wb	IHC
1	SP-C	Santa Cruz Biotechnology	13979	1:1000	
2	p53	Santa Cruz Biotechnology	#sc126	1:200	
3	P15Sp53	Cell Signaling Technology	9284	1:1000	
4	ACp53	Cell Signaling Technology	2570	1:1000	
5	Caspase-3	Abcam	ab32351	1:1000	
6	Cleaved (Cl.) caspase-3	Cell Signaling Technology	9661	1:1000	
7	PAI-1	Abcam	ab66705	1:1000	
8	β-actin	Cell Signaling Technology	3700	1:1000	
9	IL-17A	Abcam	ab79056	1:1000	1:500
10	IL-17RA	Abcam	ab180904	1:1000	
11	TRF1	Abcam	ab1423	1:1000	1:500
12	TRF2 (D1Y5D) rabbit mAb	CST	13136S	1:1000	1:500
13	β-Gal	CST	27198s	1:1000	
14	Anti-Col-1 mAb	Southern Biotech	#1340-01	1:1000	
15	Anti-FN Ab	Molecular Innovations	#ASMFBN	1:1000	
16	PNUTS	CST	14171	1:1000	
17	PP2Ac	CST	2259S	1:1000	
18	pATM	Millipore	05-740Sp	1:1000	
19	MDM2	Abcam	ab226939	1:1000	
20	ATM	Millipore	05513	1:1000	
21	Anti-αSMA mAb	Abcam	#ab5694	1:3000	
22	Tenascin-C	CST	33352	1:1000	

**Table 2:** Real-Time qPCR primers.

Primers	Forward	Reverse
IL-17A mice	ATCCCTCAAAGCTCAGCGTGTC	GGGTCTTCATTGCGGTGGAGAC
IL-17A human	ACTCCTGGGAAGACCTCATTGG	GGCCACATGGTGGACAATCG
IL-17RA mice	GTGGCGGTTTTCCCAGCCACTTTGTG	GATGCTGTGTGTCAGGTCTCCACAGT
IL-17RA human	AGACACTCCAGAACCAATTCC	TCTTAGAGTTGCTCTCCACCA
uPA	TACCGAGGAAAGGCCAACAC	TTCCCCAGGCCTAGGCTAAT
PAI-1	TCAGTGGCCAATGGAAGACC	CTGGTAGGGCAGTTCCACG

## Data Availability

The raw data supporting the conclusions of this article will be made available by the authors on request.
